# Chronological Course and Clinical Features after Denver Peritoneovenous Shunt Placement in Decompensated Liver Cirrhosis

**DOI:** 10.3390/tomography10040036

**Published:** 2024-03-25

**Authors:** Shingo Koyama, Asako Nogami, Masato Yoneda, Shihyao Cheng, Yuya Koike, Yuka Takeuchi, Michihiro Iwaki, Takashi Kobayashi, Satoru Saito, Daisuke Utsunomiya, Atsushi Nakajima

**Affiliations:** 1 Department of Diagnostic Radiology, Yokohama City University Graduate School of Medicine, Yokohama 236-0004, Japan; singo109@yokohama-cu.ac.jp (S.K.); a9613097@gmail.com (S.C.); e133047f@yokohama-cu.ac.jp (Y.T.); 2 Department of Gastroenterology and Hepatology, Yokohama City University Graduate School of Medicine, Yokohama 236-0004, Japan; nogamia@yokohama-cu.ac.jp (A.N.); yoneda@yokohama-cu.ac.jp (M.Y.); michihir@yokohama-cu.ac.jp (M.I.); tkbys@yokohama-cu.ac.jp (T.K.); ssai1423@yokohama-cu.ac.jp (S.S.); nakajima.ats.dr@yokohama-cu.ac.jp (A.N.); 3 Department of Interventional Radiology, Saiseikai Yokohama Nanbu Hospital, Yokohama 234-0054, Japan; r06118@hotmail.co.jp; 4 Department of Gastroenterology, Sanno Hospital, Tokyo 107-0052, Japan

**Keywords:** peritoneovenous shunt, decompensated liver cirrhosis, refractory ascites, interventional radiology, disseminated intravascular coagulation, heart failure

## Abstract

Background: Refractory ascites affects the prognosis and quality of life in patients with liver cirrhosis. Peritoneovenous shunt (PVS) is a treatment procedure of palliative interventional radiology for refractory ascites. Although it is reportedly associated with serious complications (e.g., heart failure, thrombotic disease), the clinical course of PVS has not been thoroughly evaluated. Objectives: To evaluate the relationship between chronological course and complications after PVS for refractory ascites in liver cirrhosis patients. Materials and Methods: This was a retrospective study of 14 patients with refractory ascites associated with decompensated cirrhosis who underwent PVS placement between June 2011 and June 2023. The clinical characteristics, changes in cardiothoracic ratio (CTR), and laboratory data (i.e., brain natriuretic peptide (BNP), D-dimer, platelet) were evaluated. Follow-up CT images in eight patients were also evaluated for ascites and complications. Results: No serious complication associated with the procedure occurred in any case. Transient increases in BNP and D-dimer levels, decreased platelet counts, and the worsening of CTR were observed in the 2 days after PVS; however, they were improved in 7 days in all cases except one. In the follow-up CT, the amount of ascites decreased in all patients, but one patient with a continuous increase in D-dimer 2 and 7 days after PVS had thrombotic disease (renal and splenic infarction). The mean PVS patency was 345.4 days, and the median survival after PVS placement was 474.4 days. Conclusions: PVS placement for refractory ascites is a technically feasible palliative therapy. The combined evaluation of chronological changes in BNP, D-dimer, platelet count and CTR, and follow-up CT images may be useful for the early prediction of the efficacy and complications of PVS.

## 1. Introduction

Refractory ascites associated with portal hypertension caused by decompensated cirrhosis is not only an important prognostic factor but also a serious problem for a patient’s quality of life [1,2,3,4]. Approximately 10% of patients with liver cirrhosis suffer from refractory ascites [5], and the mortality rate is reported to exceed 20% at 1 year [6]. Liver transplantation is the foremost long-term therapeutic option for refractory ascites associated with decompensated cirrhosis; however, it is not widely performed. In addition, several patients with advanced cirrhosis are not deemed eligible for the surgery of liver transplantation. Other therapeutic options for refractory ascites include large-volume paracentesis (LVP) with albumin substitution, indwelling peritoneal catheter placement, transjugular intrahepatic portosystemic shunt placement (TIPS), automated low-flow ascites pump implantation, and peritoneovenous shunt (PVS) placement.

PVS placement is a palliative interventional radiological therapy that involves the placement of a shunt that connects the ascites in the abdominal cavity to the vein, leading to improvements in quality of life [7]. PVS is indicated for patients with serious hepatic or malignant ascites, those whose symptoms limit their daily life, and those who are resistant to medical treatment. LVP with albumin substitution may be the first-line therapy for ascites; however, eligible patients must have an expected survival prognosis [8], and repeated paracentesis is required every 10–14 days in most patients, even if the entire volume of the ascitic fluid is drained [9,10]. An indwelling peritoneal catheter is widely used as a palliative treatment for refractory ascites [2], although a high incidence of infection was reported [11]. In Japan, it is currently not covered by insurance, and it is clinically unavailable. PVS placement, which is a feasible procedure with a high success rate [1,12], can avoid the pain associated with repeated punctures during LVP. On the other hand, PVS might be associated with potential risks of serious postoperative complications, such as disseminated intravascular coagulation (DIC), sepsis, and heart failure [2]. Understanding the chronological clinical course and risks of complications after PVS is essential for appropriate patient management after the procedure. However, the clinical features of patients with chronic liver disease who underwent PVS placement have not been thoroughly evaluated.

Thus, we aimed to evaluate the clinical features after PVS placement for refractory ascites caused by decompensated cirrhosis, especially heart failure and DIC.

## 2. Materials and Methods

### 2.1. Study Design and Patient Population

This was a single-center, retrospective, cohort study of patients with refractory ascites caused by decompensated cirrhosis at our hospital between June 2011 and June 2023. This study enrolled patients who underwent the placement of a PVS due to refractory ascites being unresponsive to pharmacotherapy or necessitating frequent LVP on a weekly basis. The exclusion criteria were as follows: severe heart failure, end-stage renal failure without the initiation of dialysis, spontaneous bacterial peritonitis, sepsis, bloody ascites, intra-abdominal adhesions, hyperbilirubinemia (bilirubin levels > 2.0 mg/dL), coagulation disorders (platelet count < 50 × 10^3^/μL, INR > 2.0), untreated esophageal varices, and gastrointestinal bleeding. Fourteen patients were enrolled in this study. The survival status of each patient was confirmed on 30 September 2023. This study was conducted in accordance with the principles of the 1975 Declaration of Helsinki and approved by the Ethics Committee of our hospital (approval number: F230700027, 8 August 2023). Informed consent was obtained in the form of the option to opt out on the website.

### 2.2. Denver Peritoneovenous Shunt Placement

Denver PVS placement was performed under general anesthesia for 5 of the 14 patients and under intravenous anesthesia for the other 9 patients. Local anesthesia was achieved in all cases using 1% lidocaine and epinephrine. A PVS placement kit (Becton Dickinson and Company, Franklin Lakes, NJ, USA), which consisted of a perforated 15.5 Fr peritoneal catheter, an 11.5 Fr venous catheter, and a two-lumen pump chamber with a non-return valve made of silicone, was used in all cases. All punctures were created under ultrasound guidance. The abdomen, chest wall, and lower neck were disinfected and covered with sterile drapes. Thereafter, the right subclavian vein was punctured with an 18-gauge needle, and a 0.038-inch guidewire was inserted into the inferior vena cava under fluoroscopic guidance. After creating skin incisions and dilating puncture routes, a 15.5 Fr peritoneal catheter was inserted into the abdominal cavity via a 16 Fr peel-away sheath. Ultrasonography was performed before the procedure to confirm that the insertion site was free of intestinal adhesions. To prevent the rapid return of ascites into the veins, 4–5 L of ascites was removed using a catheter while monitoring blood pressure. A subcutaneous pocket under the right costal arch for chamber placement and a subcutaneous tunnel to the right subclavian-vein puncture site were created. An 11.5 Fr venous catheter was passed through this tunnel; fluoroscopy was used to confirm that there was no deflection or kinking of the catheter within the subcutaneous tunnel. The puncture route was dilated, and a 12 Fr peel-away sheath was inserted. Thereafter, an 11.5 Fr venous catheter, pre-adjusted to the desired position, was placed in the superior vena cava under fluoroscopic guidance. The skin incision was sutured using absorbable sutures, and the procedure was completed.

### 2.3. Clinical Course after PVS (Heart Failure and DIC)

Data on the medical histories, physical measurements, and laboratory test results of the patients were obtained from their medical records. We retrospectively analyzed the clinical features and data trends that could be associated with heart failure and DIC. We measured the brain natriuretic peptide (BNP) level and cardiothoracic ratio (CTR) on the chest X-ray image, which are frequently used in clinical practice, to evaluate the degree of heart failure and analyzed trends of changes in these values. Platelet count and D-dimer level were measured to evaluate the degree of DIC, and trends of changes in the values were evaluated as well. Laboratory tests were performed using standard techniques. Blood samples were collected after a night of fasting, and albumin, total bilirubin, D-dimer, and BNP levels were measured before PVS and 2 and 7 days after PVS placement.

### 2.4. Chronological Changes of Ascites

In the patients who underwent follow-up CT imaging, the volume of ascites was compared between the baseline CT before PVS and follow-up CT after PVS. The volume of ascites was estimated by the 5-point method on CT images according to the previous study [13]. The average thickness of ascites of 5 points from A to E were measured on CT images as follows: A (cm), the distance between the inner surface of the right abdominal wall and the surface of the liver; B (cm), the distance between the inner surface of the left abdominal wall and the surface of the spleen; C (cm), the posterior pole of the right paracolic gutter; D (cm), the posterior pole of the left paracolic gutter; E (cm), the distance between the inner surface of the anterior abdominal wall and the line though the bilateral femoral arteries. The amount of ascites was calculated by the following equation: (A + B + C + D + E) × 200 (mL).

### 2.5. Incidence of Complications and PVS Patency

PVS complications (i.e., procedure-related complications, acute heart failure, DIC, thrombotic disease) and long-term PVS patency were recorded in the clinical course.

### 2.6. Statistical Analysis

Data were analyzed using the JMP software (version 17.0; SAS Institute Inc., Cary, NC, USA). Continuous and ordinal variables were compared using the unpaired *t*-test and are expressed as means (SD). Survival probability was illustrated using the Kaplan–Meier curve.

## 3. Results

### 3.1. Patient Characteristics

The baseline characteristics of the participants are presented in Table 1. The underlying liver disease in three, two, six, and three of the patients was hepatitis C, autoimmune hepatitis, metabolic dysfunction-associated steatotic liver disease, and others, respectively. Hepatic encephalopathy was observed in no patients. It was difficult to ascertain the duration of cirrhosis and the period of ascites because our patients were referred from other institutions and their precise information regarding the duration of cirrhosis and the period of ascites was limited before the referral.

### 3.2. Chronological Changes of Clinical Course

#### 3.2.1. Heart Failure Index before and after PVS (BNP, CTR)

**Figure 1:**
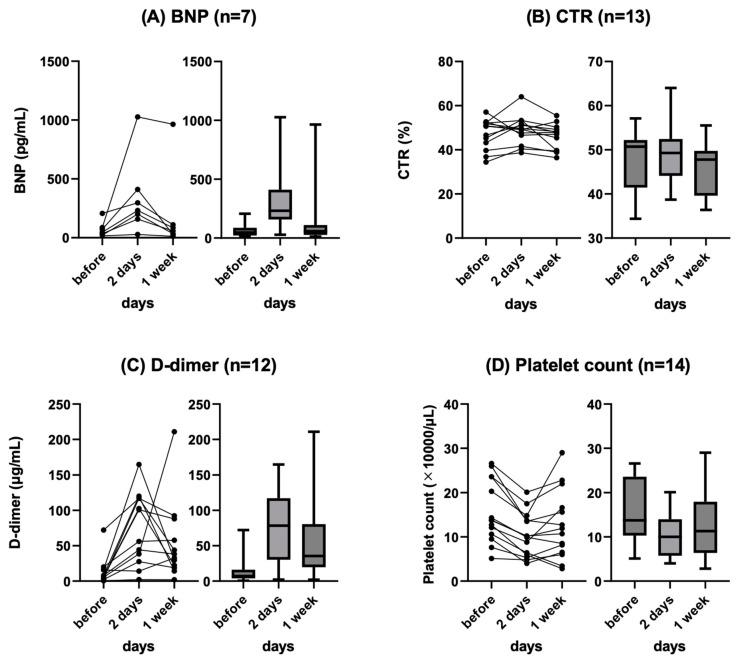
Changes in BNP (**A**), CTR (**B**), D-dimer (**C**), and platelet (**D**) at baseline, 2 days, and 1 week after PVS placement. Line chart (left) and box plots (right) are shown. BNP: brain natriuretic peptide, CTR: cardiothoracic ratio, PVS: peritoneovenous shunt.

The change in BNP level before and after (2 and 7 days) PVS placement was measured in seven patients. The mean BNP level before and 2 and 7 days after PVS placement was 68.9 pg/mL, 336.1 pg/mL, and 184.1 pg/mL, respectively, and the mean CTR was 47.2%, 49.0%, and 46.0%, respectively. In all patients, BNP level tended to show transient deterioration within 2 days after PVS placement but recovered within 1 week. The details are shown in Figure 1A.

The change in CTR before and after (2 and 7 days) PVS placement was measured in 13 patients. The mean CTR before, 2 days after, and 7 days after PVS placement was 47.2%, 49.0%, and 46.0, respectively. CTR showed a tendency to transiently worsen after PVS placement but spontaneously recovered in all patients. The details are shown in Figure 1B.

#### 3.2.2. Thrombotic Disease Index before and after PVS (D-Dimer, Platelet Count)

The change in D-dimer level before and after (2 and 7 days) PVS placement was measured in 12 patients. The mean D-dimer level before, 2 days after, and 7 days after PVS placement was 13.6 ng/mL, 75.5 ng/mL, and 54.3 ng/mL, respectively. All patients showed a tendency for transient worsening after PVS placement, and all patients except one recovered spontaneously. One patient with a complication of thrombotic disease (renal and splenic infarction) showed a continuous increase in D-dimer levels 2 and 7 days after PVS placement. The details are shown in Figure 1C.

The change in platelet count before and after (2 and 7 days) PVS placement was measured in all patients (*n* = 14). The mean platelet counts before and 2 and 7 days after PVS placement was 156 × 10^3^/µL, 104 × 10^3^/µL, and 126 × 10^3^/µL, respectively. All patients showed a trend toward decreased platelet count after PVS placement and all spontaneously recovered. The details are shown in Figure 1D.

#### 3.2.3. Ascites before and after PVS

CT scans were conducted before PVS placement in 10 of 14 patients, and among them, follow-up CT scans after PVS were performed in 8 patients. CT volumetry of the ascites showed that the mean volume of ascites was 3390 mL (range: 1920 to 4720 mL) before PVS and 928 mL (range: 0 to 1560 mL) after PVS.

In 2 out of the 14 cases, several abdominal paracenteses were required even after PVS placement, while in 12 cases, paracentesis was not performed during the period of shunt patency (Table 2). The use of diuretics was reduced after PVS placement (Table 2).

### 3.3. Complications and PVS Patency

**Figure 2:**
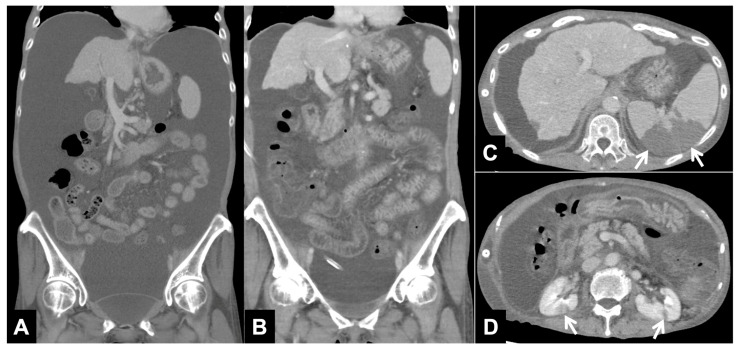
CT images with continuous elevation of D-dimer (Patient No. 12 in Table 2). (**A**) Reconstructed coronal CT image before PVS placement shows a large amount of ascites. (**B**) Reconstructed coronal CT image one week after PVS placement shows a decrease in ascites. (**C**,**D**) Axial CT images one week after PVS placement shows splenic infarction ((**C**) arrows) and bilateral renal infarction ((**D**) arrows).

No serious complication associated with the PVS procedure occurred. In the clinical course, none of the patients had overt heart failure or DIC during the study period, but vascular thrombus and associated splenic infarction and bilateral renal infarction were observed in the patient with the persistent elevation of D-dimer 2 and 7 days after PVS placement (Figure 2). In this patient, a notable reduction in ascites was achieved, and the shunt remained patent. Infection was observed in 2 (14.3%) of the 14 patients who were treated with standard antibiotic therapy.

**Figure 3:**
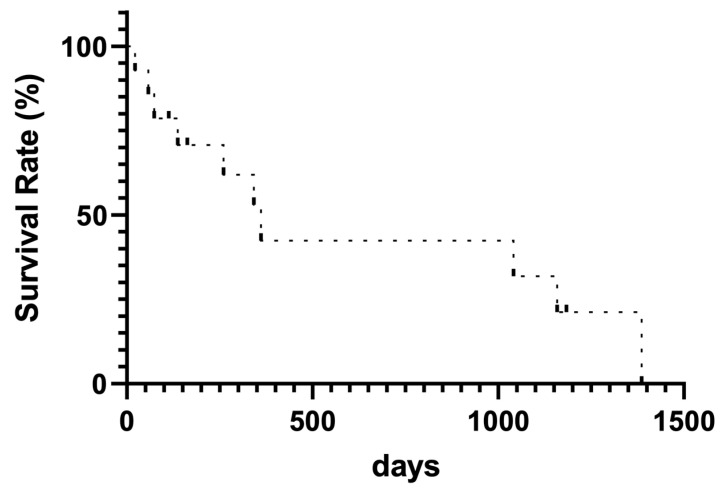
Kaplan–Meier curve for survival rate.

In the long-term follow-up, PVS occlusion was observed in 6 of 14 patients (42.8%) using fluoroscopy and color Doppler ultrasound (Table 2). Of these, three patients required system replacement because of thrombus obstruction of the PVS catheter lumen (*n* = 5) and catheter kinking (*n* = 1). The mean duration of PVS patency was 345.4 (22–1386) days, and 10 patients died. The median survival after PVS placement was 474.4 (22–1386) days (Figure 3).

## 4. Discussion

In this retrospective observational study, we evaluated the chronological course of heart failure and DIC in patients who underwent PVS placement for refractory ascites caused by decompensated liver cirrhosis. The procedure was successfully performed in all cases, and none of the patients had any major procedure-related complications. The results showed that although an increase in BNP level and the worsening of CTR occurred 2 days post-PVS placement, these changes were mild and transient and did not result in heart failure. Most patients also showed transiently elevated D-dimer levels and reduced platelet counts, which could be primarily attributed to increased fibrinolysis and blood dilution following the influx of ascitic fluid. The transient exacerbation in laboratory data results and CTR did not lead to significant signs of heart failure or DIC. On the other hand, the patient showing a continuous increase in D-dimer from 2 to 7 days after PVS had thrombotic diseases, i.e., renal and splenic infarction. The continuous exacerbation of laboratory data results 2 and 7 days after PVS might be a clue to identify the complication of PVS, and follow-up imaging studies such as chest X-ray and CT imaging should be considered, although transient abnormality might be just a reaction of PVS, suggesting a positive postoperative clinical course.

The usefulness of PVS placement for the management of patients with serious cirrhosis was first reported by Leveen et al. in 1974 [14]. Despite limited evidence, approximately 80% of patients experience continuous symptom relief after PVS placement, with reported benefits ranging from renal function improvement to reduced reliance on diuretics [12,15,16]. PVS is also advantageous for the patient’s quality of life because repeated punctures are not necessary to control the ascites. However, PVS has not been established as the first-choice therapy for ascites, mainly due to its complications such as volume overload, DIC, and heart failure [2]. In consideration of these unfavorable outcomes, major liver associations, such the American Association for the Study of Liver Diseases and the European Association for the Study of the Liver, have limited recommendations for PVS placement [3,17,18]. PVS placement is only considered in cases of refractory ascites that is difficult to control even with LVP or continuous ascites removal therapy [4]. TIPS was reported to be indicated for patients with refractory ascites who require frequent LVP [19], and it was suggested to have a long-term efficacy compared to PVS placement [20]. However, in Japan, the insurance coverage of other shunt techniques (e.g., TIPS) and the availability of liver donors is limited; therefore, the potential necessity of PVS placement has been relatively higher than in Western countries [17,18,21]. Our study results showed the adequate safety and efficacy of PVS. We posit that our favorable results could be attributed to the substantial drainage of ascitic fluid during the PVS placement procedure, and PVS might be more widely performed in the management of severe cirrhosis patients with refractory ascites, although further studies with larger populations should be conducted.

None of the patients in the present study showed acute heart failure nor DIC after PVS. The potential of PVS to induce decompensated heart failure has been acknowledged; thus, thorough cardiac evaluation before the procedure is necessary. Heart failure associated with PVS placement, which is reported to occur in 3–16% of cases [12,22,23], is attributed to cardiovascular strain caused by the rapid inflow of ascitic fluid into blood vessels. We consider that our PVS technique with the drainage of ascitic fluid during the procedure helped the prevention of heart failure exacerbation. The incidence of DIC after PVS placement reportedly ranges from 0% to 35% [12,23,24,25]. Risk factors for DIC after PVS placement include bloody ascites and hyperbilirubinemia, with bilirubin levels ≥ 2 mg/dL identified as potential triggers, and our patients did not have these risk factors [8]. This highlights the importance of appropriate patient selection and the management of the inflow of ascitic fluid into vessels during PVS placement. Shunt occlusion was observed in approximately 40% of the patients during the follow-up period in this study. This finding is consistent with the results of previous studies, which indicated high incidences of shunt occlusion ranging from 16% to 45% [12,22,26,27]. The mean duration of PVS patency in the present study was 345 days, with a low incidence of early occlusion. In the patients with encapsulated ascites or intra-abdominal adhesions, the inability to adequately drain ascites may be considered to increase the risk of shunt occlusion. In such a case, it is deemed contraindicated [8]. The thrombotic obstruction of the catheter lumen was a major cause of PVS occlusion. One of the 12 patients showed increased D-dimer levels from 2 to 7 days after PVS placement and had the major complication of thrombotic infarction of the kidneys and spleen, although the PVS per se was patent. The relationship between the continuous increase in D-dimer level and early shunt occlusion should be investigated in the future studies.

This study has several limitations. First, this was a retrospective study with a small patient population in a single center. In addition, the optimal timing for evaluating post-PVS placement data is unclear. We plan to accumulate more patients with PVS in several hospitals and conduct further studies. Second, although physical improvements were confirmed in this study, the subjective evaluation of improvement in quality of life using a questionnaire was not conducted. The efficacy and value of PVS placement for patients with refractory ascites should be comprehensively assessed. Third, we did not compare the use of different treatment modalities (e.g., Denver shunt, Le Veen shunt, or sapheno-peritoneal shunt) and surgical methods.

## 5. Conclusions

This study demonstrated that simultaneous PVS placement and the complete drainage of ascites could be safe and effective for the management of refractory ascites associated with decompensated cirrhosis. The combined evaluation of chronological changes in BNP, D-dimer, platelet count, and CTR 2 and 7 days after PVS and follow-up CT images may be useful for the early prediction of the efficacy and complications of PVS.

## Figures and Tables

**Table 1:** Baseline patient characteristics.

	Patients (*N* = 14)
Age (years, SD)	66.2 (15.6)
Male sex (n, %)	8 (57.1)
Height (m, SD)	1.59 (0.02)
Body Weight (kg, SD)	67.2 (5.0)
HCV/AIH/SLD/others	3/2/6/3
HCC (%)	3 (21.4)
Albumin (g/dL, SD)	2.77 (0.23)
Total Bilirubin (mg/dL, SD)	1.46 (0.28)
D-dimer (μg/mL, range)	14.8 (0.5–72.2)
BNP (pg/mL, range)	78.9 (7.3–300.2)

Data are presented as mean values with SDs. AIH, autoimmune hepatitis; BNP, brain natriuretic peptide; HCV, hepatitis C virus; HCC, hepatocellular carcinoma; SD, standard deviation, SLD, steatotic liver disease.

**Table 2:** Details of patients’ progress.

No.	Age	Etiology	Existence date	PVS Occlusion	Ascites Volume		Abdominal Paracentesis (Times/Month)		Diuretic						**Lifetime Date**	**Cause of Death**
									Before			After				
					Before	After	Before	After	F	S	T	F	S	T		
1	60s	Extrahepatic PVO	864	Yes	3600	960	2	0	140	100	–	40	100	–	1159	Colon cancer
2	60s	AIH	23	Yes	N/A		3	0	60	–	7.5	60	–	15	58	LF
3	60s	SLD	1386	No	N/A		3	0	40	–	3.75	80	50	7.5	1386	LF
4	70s	AIH	74	Yes	N/A		4	2	20	–	3.75	20	–	3.75	74	LF
5	70s	SLD	212	Yes	4140	820	2	0	80	25	7.5	–	25	–	1041	Infection
6	60s	SLD	1184	No	1920	1420	3	0	–	–	–	–	–	–	–	–
7	60s	SLD	133	No	N/A		3	0	20	–	7.5	–	–	–	137	LF
8	70s	HCV	22	Yes	N/A		4	0	40	50	7.5	40	–	–	22	CI
9	60s	HCV	62	yes	2900	0	3	0	80	50	7.5	80	50	7.5	260	GB rupture
10	60s	HCV	361	No	2500	1480	4	0	40	–	7.5	20	–	7.5	361	SBP
11	70s	SLD	38	No	N/A		4	2	40	100	7.5	10	50	7.5	342	CHF
12	70s	PBC	342	No	4720	1560	3	0	20	50	7.5	0	50	7.5	–	–
13	40s	SLD	22	No	3160	0	3	0	40	100	7.5	20	–	3.75	–	–
14	40s	SLD	113	No	4180	1180	4	0	40	100	7.5	20	100	7.5	–	–

AIH, autoimmune hepatitis; CI, cervical infarction; F, Frosemide, LF, liver failure; HCV, hepatitis C virus; PBC, primary biliary cholangitis; PVO, portal vein obstruction; PVS, peritoneovenous shunt; S, Spironolactone; SBP, spontaneous bacterial peritonitis; SLD, steatotic liver disease; T, Tovaptan.

## Data Availability

The data underlying the case series are stored in an internal database.
